# The Role of Peptides in the Design of Electrochemical Biosensors for Clinical Diagnostics

**DOI:** 10.3390/bios11080246

**Published:** 2021-07-23

**Authors:** Patrick Severin Sfragano, Giulia Moro, Federico Polo, Ilaria Palchetti

**Affiliations:** 1 Department of Chemistry “Ugo Schiff”, University of Florence, Via della Lastruccia 3, 50019 Sesto Fiorentino, Italy; patrickseverin.sfragano@unifi.it; 2 Department of Molecular Sciences and Nanosystems, Ca’ Foscari University of Venice, Via Torino 155, 30172 Venice, Italy; giulia.moro@unive.it (G.M.); federico.polo@unive.it (F.P.)

**Keywords:** peptide, electrochemical biosensor, antifouling, protease, biomarker, bioreceptor

## Abstract

Peptides represent a promising class of biorecognition elements that can be coupled to electrochemical transducers. The benefits lie mainly in their stability and selectivity toward a target analyte. Furthermore, they can be synthesized rather easily and modified with specific functional groups, thus making them suitable for the development of novel architectures for biosensing platforms, as well as alternative labelling tools. Peptides have also been proposed as antibiofouling agents. Indeed, biofouling caused by the accumulation of biomolecules on electrode surfaces is one of the major issues and challenges to be addressed in the practical application of electrochemical biosensors. In this review, we summarise trends from the last three years in the design and development of electrochemical biosensors using synthetic peptides. The different roles of peptides in the design of electrochemical biosensors are described. The main procedures of selection and synthesis are discussed. Selected applications in clinical diagnostics are also described.

## 1. Introduction

Peptides are sequences of amino acids of varying length and weight. Only 20 of the hundreds of known amino acids account for the vast majority of residues that make up human proteins [1]. The chemical structure of amino acids that occur in protein varies only in the R-group at the carbon in alpha position, Cα, and are referred to as α-amino acids [2]. Not all biologic amino acids are α-amino acids; β-amino acids (e.g., β-taurine), as well as γ-amino acids (e.g., γ-aminobutyric acid), also play important biochemical roles. α-amino acids are chiral, with the exception of glycine, and mainly occur in the l-form, even though small quantities of D-amino acids occur in biological fluids but without specific function. D-serine is an exception and serves as a neurotransmitter in cerebrospinal fluid. Isoleucine and threonine have a second asymmetric carbon that gives rise to stereoisomers. Furthermore, a number of rare amino acids are recovered from protein hydrolysates, in addition to the 20 protein-forming amino acids.

The chemical behaviour of the R group dictates the chemical behaviour of each α-amino acid, which can be categorized as charged, hydrophilic, or hydrophobic. The hydrophobic amino acids with aliphatic residues are alanine (A), isoleucine (I), leucine (L), methionine (M), and valine (V). Amino acids with aromatic residues such as phenylalanine (F), tryptophan (W), and tyrosine (Y) are also hydrophobic [3]. The aliphatic residues generally provide a hydrophobic environment whereas aromatic residues are usually involved in π–π stacking. The hydrophilic, polar residues participate in hydrogen bonding either via OH (serine (S) and threonine (T)) or CONH_2_ groups (asparagine (N) and glutamine (Q)). The ionisable residues can be positively charged, (histidine (H), lysine (K), and arginine (R)) or negatively charged, such as in aspartic acid (D) and glutamic acid (E). Other natural amino acids are cysteine (C), glycine (G), and proline (P). Proline exhibits hydrophobic behaviour, whereas cysteine, with its thiol side chain, is an important source of sulfur in human metabolism.

Peptides adopt specific conformations depending on the position of each R-group in the amino acid sequence. The secondary structure of peptides (α- helices, β-sheets, and β-hairpins) is driven by noncovalent intermolecular interactions, such as hydrogen bonding, van der Waals forces, π-stacking, and hydrophobic and electrostatic interactions. The secondary structure of peptides can be modulated by introducing modifications in the amino acids, thus favouring interactions with other peptides or proteins. Amino acids can be considered as natural molecular building blocks that spontaneously arrange themselves to form highly organized structures with well-defined functional properties [2].

Recently, peptides have been proposed to be interesting and versatile tools for the design and development of biosensors [3,4,5,6]. Peptides can be easily obtained by chemical synthesis methods, avoiding the need for in-vivo, laborious procedures, as in the case of antibodies. Moreover, they are also biocompatible. Notably, research activity concerning peptide-based biosensors and bioassays is increasing, and, at the time of writing, around 3000 matches are found when searching for the keywords “peptide” and “biosensor” in commonly employed search engines (SciFinder, CAS).

Electrochemical biosensors are powerful tools that allow for cost-effective, easy, and fast detection and/or monitoring of analytes in clinical diagnostics [7,8,9,10,11,12], and are often employed in the framework of point-of-care testing (PoCT). Peptides represent a promising class of affinity biorecognition elements that can be coupled to electrochemical transducers. The benefits lie mainly in their stability and selectivity toward a target analyte. Furthermore, they can be synthesized rather easily and modified with specific functional groups, thus making them suitable for the development of novel architectures for biosensing platforms, as well as alternative labelling tools. Also, peptides have been proposed as antibiofouling agents. Indeed, biofouling caused by the accumulation of biomolecules on electrode surfaces is one of the major issues and challenges to be addressed in the practical application of electrochemical biosensors.

**Figure 1:**
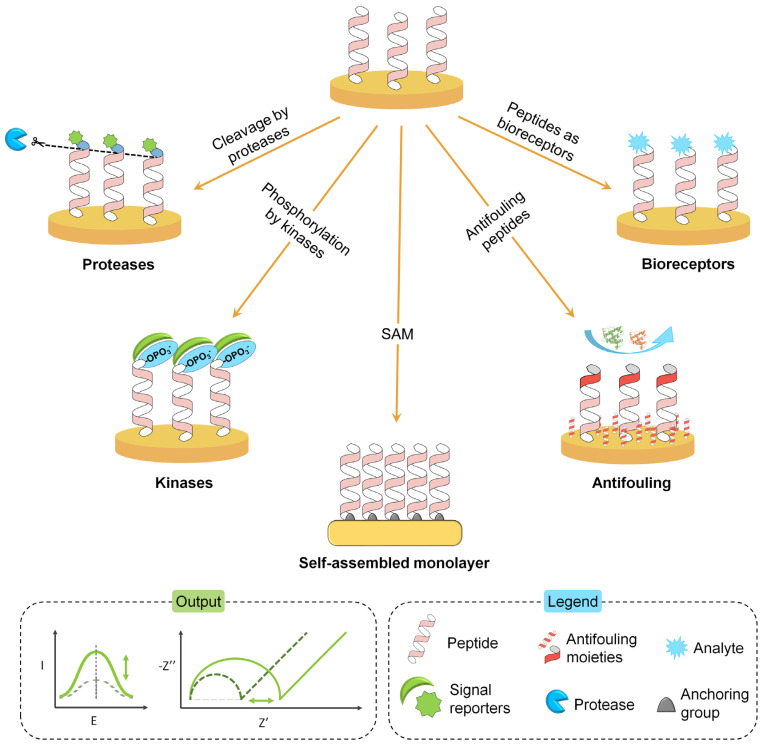
Schematic summary of the different roles of synthetic peptides in biosensors, as discussed in this review. Proteases can cause the cleavage of specific peptide sequences; kinases phosphorylate the hydroxyl groups in the peptide sequence; furthermore, peptides can form self-assembled monolayers and work as antifouling agents and biorecognition elements. The output can be an increase (signal-on) or a decrease (signal-off) of the electrochemical readout. Both label and label-free approaches are possible in biosensing. Picture not to scale.

Herein, we summarise trends from the last three years in the design and development of electrochemical biosensors using synthetic peptides. In comparison with other recent review papers mainly focused on protein detection [13] or on electrochemical methods in peptide-based assays [3], the different roles of peptides in the design of electrochemical biosensors are described here—from their use as antifouling agents to their role in the development of catalytic, as well as affinity, electrochemical biosensors. Moreover, the main procedures of selection and synthesis are discussed. Figure 1 shows a schematic summary of the various applications of peptides described throughout the review. Regarding detection, both label and label-free approaches are possible and different electrochemical detection methods are achievable using three main quantities: current (amperometric and voltammetric biosensors), potential, and impedance. Nonelectroactive peptides can be successfully combined with impedance/voltammetric transducers [14,15]. Furthermore, peptides can be easily labelled with redox-active probes, such as ferrocene (Fc) or methylene blue [16,17,18] for signal-on/signal-off detection. Selected applications in clinical diagnostics, in which these kinds of detection strategies are exploited, are discussed in the following sections.

## 2. Peptide Selection, Synthesis, and Characterisation

The design of successful peptide-based sensing platforms requires accurate selection, synthesis, and characterisation of the bioreceptor to optimize the surface coverage, while assuring and maximizing interaction with the target, and, to guarantee high-affinity, selective and reproducible recognition [19]. Ligand peptide selection can be challenging if one considers the commercial availability of hundreds of amino acid building blocks (naturally occurring and not) and the different folding of the final sequence (linear, cyclic, and bicyclic). Despite this high variability, the chemical synthesis of peptides can be easily implemented with great rapidity and high production rates. This aspect is of particular interest, as the availability of well-grounded and straightforward synthetic protocols allows several sequences to be tested in a short time, thus making these biomolecules highly competitive in pharmaceutical, as well as sensing, applications [20]. Therefore, genetically encoded peptide ligands with tailored properties can be generated by screening large combinatorial libraries using in-vitro display techniques [21]. Phage display was the first combinatorial technique used for the direct evolution of peptides, as described by Smith in 1985 [22]. The identification of high-affinity sequences is achieved by screening a wide library of random peptides that are displayed on the surface of the phage against the target of interest, such as a small molecule, a protein, etc. The displayed peptides, representing the phenotype, have a physical linkage with their encoding DNA or RNA sequences, the genotypes. After exposure to the target, only the binding peptides are sequenced via mass spectrometry-based methods to obtain the amino acid sequences [23]. To enhance binding affinity and to avoid nonspecific binding and blocking side-phenomena, phage display was further implemented in recent decades [24]. A similar selection mechanism also characterised other well-known combinatorial techniques described in the 1990s: from mRNA display [25] to ribosome display [26], bacteria display [27], and yeast display [28,29]. These experimental approaches can be integrated by computational ones from in-silico evolution and structural bioinformatics to atomistic simulations, as recently discussed by D’Annessa et al. [30]. Xiao et al. described the successful selection of peptides for biosensing applications using solely in-silico evolution [31]. This example underlines the wide variety of paths available for peptide selection. Regardless of the approach chosen for the selection, prior to proceeding with large-scale synthesis of the selected sequences, an in-depth characterisation of the peptide–target complex is required. The study of the binding kinetics and thermodynamics, along with the structural features of the peptide–target complex, is instrumental in evaluating the applicability of the selected peptide as a bioreceptor and, eventually, the identification of the most suitable design for the sensing platform. The design of the peptide–target characterisation protocol should take into account the final application of the peptide sequence (i.e., the biosensing platform) as the binding performance can vary depending on the physical and chemical properties of the surrounding environment, as recently discussed for other biorecognition elements [32]. Characterisation of the peptide–target complex is therefore fundamental to the evaluation of the peptide’s applicability, the discrimination of a restricted pool of selected sequences, and, eventually, the consideration to improve biorecognition elements, for instance, by using peptides in conjugation with other biomolecules [33,34]. Once the peptide bioreceptor is characterised, it is possible to proceed with its synthesis following different approaches, such as solution-based [35] or solid-phase peptide synthesis (SPPS) [36], compatible with batch and continuous-flow reactors [37]. The possibility of carrying out peptide synthesis within partially or fully automated systems guarantees the ease and rapidity of synthesis and large-scale applicability of these biomolecules. The synthesis is coupled with a final purification step, usually performed via reversed-phase high-performance liquid chromatography (RP-HPLC) before checking the molecular mass by mass spectrometry.

## 3. Peptides as Self-Assembled Layers

Peptides can be immobilized at noble metal surfaces by spontaneous chemisorption processes forming ordered two-dimensional supermolecular systems known as self-assembled monolayers (SAMs). Covalent anchoring on gold surfaces is achieved mainly via gold–sulfur interaction using nonpeptide synthetic thiols or the natural thiol-containing amino acid cysteine [38,39,40]. Self-assembling peptides (SAPs) are tuneable building blocks compatible with numerous sensing strategies and nanosized biomaterials, as highlighted by the examples reported in the following sections. Heterogeneous SAMs can also be formed by mixing different biomolecules, in particular peptides and aptamers, allowing for the design of highly sensitive platforms [41]. An interface composed of a redox-tagged peptide, integrated with receptive aptamers, was developed to target C-reactive protein (CRP), a biomarker of systemic inflammation [42]. The study was further extended by designing peptides that could attach to antibodies instead of aptamers and achieve good sensitivity for clinically relevant levels of CRP [16]. By means of SPPS, both works produced and employed the Fc-tagged peptide sequence EAAC-NH_2_, in which the cysteine residue was used to obtain covalent anchoring onto a Au electrode. The resulting SAM shows high surface density and a well-ordered structure.

Other known immobilization procedures can be used to develop peptide-based biosensors, namely physical adsorption (based on the noncovalent interaction between peptides and the transducer surface), the entrapment into a three-dimensional network of gel or polymers, as well as covalent chemical binding via functional moieties and selected reagents such as carbodiimide and hydroxysuccinimide derivatives [43].

## 4. Peptides as Antifouling Agents

Modification of the electrode surface may occur during electrochemical measurement in clinical specimens due to nonspecific binding and an accumulation of the products derived from electrochemical reactions or the components of the complex matrix. The nonspecific adsorption of proteins, cells, or micro-organisms on electrode surfaces is a major concern in biomedical applications, as well as the electrochemical oxidation of phenolic compounds that form radicals and therefore polymeric structures, which are insoluble and precipitate from the solution onto the electrode surface. 

The layer of these modifiers impedes the direct contact of any electroactive compound with the electrode surface, thus hampering electron transfer and diminishing the electrochemical signal. This phenomenon is known as the fouling process [44,45] and it significantly affects the sensitivity and reproducibility of electrochemical biosensors. Several physicochemical causes are involved in the fouling process, which depend on the chemical/biochemical species involved and the nature of the electrode surface. These include electrostatic interactions and hydrophobic and intermolecular forces. Proteins, for instance, generally tend to adsorb onto hydrophobic interfaces rather than onto hydrophilic ones.

Antifouling procedures and treatments are therefore required to improve the reliability, stability, and performance of electrochemical biosensors, especially when clinical specimens are analysed. To date, approaches to enhance antifouling resistance can be classified as physical treatments, which alter the surface morphology, and chemical treatments, which immobilise selected reagent monolayers on the electrode surface. Whitesides and coworkers proposed four molecular-level characteristics for the evaluation of protein-resistant monolayers: (1) the presence of polar functional groups, i.e., hydrophilicity, (2) the presence of hydrogen bond acceptor groups, (3) the absence of hydrogen bond donor groups, and (4) the absence of net charge [46].

Thus, several bio-inert molecules have been developed or employed that effectively decrease the adsorption of protein or other biomolecules on electrode surfaces, including poly(ethyleneglycol)s, polyglycerols, zwitterionic polymers, and polysaccharides [47]. Among these molecules, the antifouling property of hydrophilic, nonionic molecules is due to hydrogen bonding-induced surface hydration. Furthermore, strongly ionic solvation can also generate surface hydration and thus form a tightly bound water layer, which provides a physical and energetic barrier to prevent protein adsorption and cell adhesion to surfaces [48]. Peptides are claimed to be interesting antifouling materials because, apart from their easy and cost-effective synthesis, they are composed of natural amino acids which are biocompatible zwitterionic molecules and possess high hydrogen bond-donor/acceptor abilities due to the carbonyl, amine, and hydroxyl groups in the peptide backbone and side chains.

A zwitterionic peptide composed of alternating negatively charged glutamic acid (E) and positively charged lysine (K) residues which exhibits antifouling abilities and a well-defined secondary structure for closer packing of the monolayer due to a proline (P) linker peptide (PPPP) was designed by Nowinski et al. [49]. These peptide sequences have been proposed in many different SAPs, obtaining hierarchical assemblies characterized by: an interfacial region that operates as a biomolecular recognition layer or as an enhancer of the target signal [50], an antifouling region that stabilises the SAM via intermolecular interactions, and a surface anchoring portion [49], mainly using a cysteine (C) residue.

An inverted Y-shaped peptide was recently used for COVID-19 nucleic acid electrochemical detection in complex biological media [51]. The peptide consists of one antifouling main chain (KEPPPPKEKEKEKEK-biotin) in the upper part, in which the four proline residues (PPPP) assist in the formation of a stable secondary structure, and two anchor-antifouling side chains (CKEKEKEKE) in the lower part. Two (C) residues at the ends of the two side chains were used as the surface anchor to immobilise the peptides onto the electrode surface. Hierarchical peptide brushes composed of the zwitterionic antifouling peptides (CPPPPEKEKEKEK) and short-chain peptides (CPPPPEKEKEK), with the possibility of tethering more water molecules than other structures, have been proposed for the electrochemical determination of alpha-fetoprotein in serum by using an aptamer as a selective bioreceptor [52].

Many examples of multifunctional all-in-one SAPs combining anchoring, antifouling, and recognizing functions are reported in the literature [3,41,53,54,55,56,57,58]. For instance, a Y-shaped peptide (CPPPPEK (HWRGWVA)EKEKE) with both recognizing and antifouling branches was designed and attached to the electrode surface via a gold–sulfur bond to construct a biosensor for IgG determination in human serum. One of the two zwitterionic branches of this Y-shaped peptide was designed with a (EKEKEKE) sequence for antifouling, and the other one with a (HWEGWVA) sequence for recognizing the IgG [59].

Other peptide sequences with antifouling properties have recently been designed and used in the development of electrochemical sensors [60,61,62,63].

## 5. Peptides as Substrate and Signal Development Agents in Catalytic Biosensors

Enzymes represent valid biomarkers to assess the status or the progression of a variety of diseases and health conditions. Indeed, the catalytic activity of an enzyme can be used for its detection when a specific substrate is present. In peptide-based biosensors, such enzymatic substrate can be a (synthetic) peptide sequence; instead of capturing the target analyte, the peptide is used to transform the catalytic activity of the enzyme into a detectable electrochemical signal. The most important enzymes that are currently detected using peptide-based biosensors are proteases and kinases.

Proteases have potential diagnostic relevance due to their ability to hydrolyse specific peptide bonds, causing proteolysis. The proteolytic activity of proteases induces the cleavage of the peptide sequence used as substrate; in order to convert this event into a measurable signal, such peptides are usually conjugated with a signal reporter, such as a redox probe [64]. The cleavage of this labelled sequence portion provides either an increase (signal-on mechanism) or a decrease (signal-off mechanism) of the electrochemical output. In this respect, it has been shown that signal-on sensors are less susceptible to false positives [65,66]. The redox reporter can be located at the N-terminus or at the C-terminus of the amino acid sequence. In general, the choice of its location is determined by the synthetic process used (SPPS, etc.), the structural characteristics of the sequence, and the design of the sensing platform. The most common redox labels are Fc, horseradish peroxidase (HRP), and methylene blue, together with signal-enhancing nanocomposites based on reduced graphene oxide (rGO) or (noble) metals such as gold, silver, and copper. Moreover, the insertion of a flexible spacer (i.e., polyethylene glycol (PEG)) within the redox reporter seems to enhance the redox signal, promote enzyme accessibility, and confer flexibility to the probe. In this regard, Bradley and coworkers presented a systematic study evaluating the effect of different PEG-based spacers on the performances of peptide-based biosensors, highlighting their pivotal role [67]. 

Label-free approaches, in which a change of the system’s impedance is correlated to the concentration of the analyte, are also employed, although less frequently. 

One of the most investigated proteases is a family of endopeptidases, namely matrix metalloproteinases (MMPs), which have an important clinical application (when upregulated) in the identification of various pathological and physiological conditions such as neurodegenerative and cardiovascular disorders, diabetes, sepsis, arthritis, angiogenesis, and others. Moreover, as matrix-degrading enzymes, MMPs can attack and degrade various extracellular protein components of cell membranes and the extracellular matrix, and hence play a significant role in the progression, invasion, and metastasis of tumours [68]. The expression of MMPs is thus considered a good parameter to monitor the occurrence and prognosis of neoplasia. Cleavage-based electrochemical biosensors that use peptides as substrate for the proteolytic activity of MMPs constitute a booming research field due to their advantages: low cost, high selectivity, and applicability in PoCT. 

Among MMPs, MMP-2 is one of the most investigated using the abovementioned biosensors, usually employing the peptide sequence PLGVR as the proteolytic target operated by MMP-2. However, other MMPs, such as MMP-7 [69] and MMP-14 [70], are also attractive clinical-related biomarkers.

Often, the current intensity measured by these protease-based platforms is quite low. One of the main challenges of modern biosensor technology is to achieve ultrasensitivity so as to detect analytes present at extremely low concentrations. Therefore, some form of signal amplification is highly desirable. Nanomaterials are widely used to enhance the performances of biosensors; for instance, by increasing the electrochemically active surface area and improving mass transport while leading to better electrocatalytic activity and fast heterogeneous electron transfer kinetics [71]. An interesting example is the modification of the electrode surface with carbon nanotubes (CNTs) and electrochemically synthesized Au nanoparticles (AuNPs), which led to the sensitive monitoring of the activity of MMP-7 in a label-free approach [72]. The electrode surface was further functionalized with a peptide (JR2EC) that presented two binding sites for MMP-7. Its MMP-mediated hydrolysis was monitored by means of differential pulse voltammetry (DPV), achieving a limit of detection (LOD) of 6 pg/mL. The sensor was successfully tested in human serum and synthetic urine.

Nanozymes also represent an attractive alternative for the attainment of remarkable signal amplification along with stability, easy development at low cost, and resistance to acids and alkalis [73,74]. Inspired by these characteristics, Xi et al. [75] fabricated an H_2_O_2_-free peptide-based biosensor for the detection of MMP-2 using a Au@Pt bimetallic nanozyme as a signal amplification label. A peptide including the sequence PLGVR allowed detection of the protease. DPV measurement showed good linear correlation of the current intensity against MMP-2 concentration, achieving a LOD of 0.18 ng/mL. 

**Figure 2:**
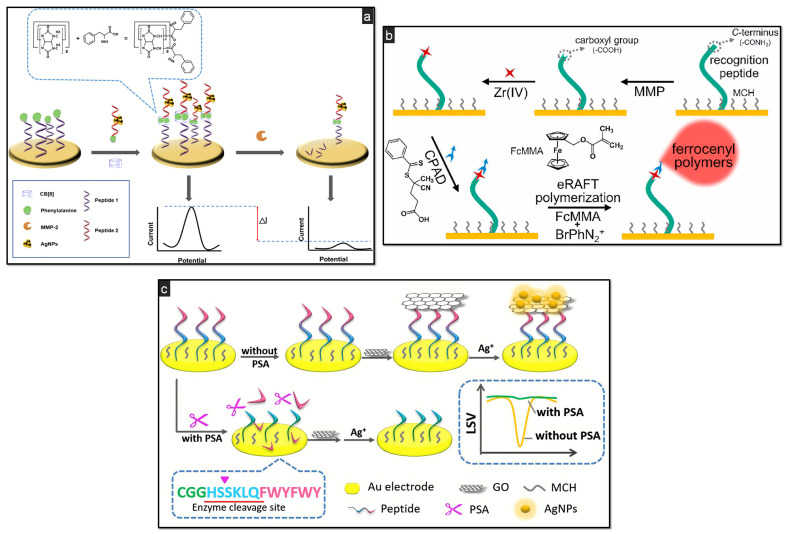
Schematic illustrations of different protease-based catalytic biosensors: (**a**) signal-amplified detection of MMP-2 through anodic stripping of AgNPs, using CB [8] as macrocyclic receptor to bind two aromatic amino acid residues via host–guest interactions, reprinted with permission from [76]; (**b**) signal-on MMP’s activity detection using a carboxyl group-free recognition peptide as proteolytic substrate, immobilised via Au–S self-assembly. Grafting of Fc polymers through eRAFT polymerization of ferrocenylmethyl methacrylate (FcMMA), reprinted with permission from [65]; (**c**) GO/AgNPs nanocomposite for the determination of PSA on Au electrode by means of linear sweep voltammetry (LSV), reprinted with permission from [83].

Other signal amplification strategies have been used to enhance the biosensor’s performance and to detect MMP-2. An appealing case is the anodic stripping of silver nanoparticles (AgNPs) on the electrode surface. In particular, a first peptide presenting a proteolytic motif sequence for the specific cleavage by MMP-2 was immobilised onto the surface of a Au electrode through a Au–S chemical bond, thus forming a SAM [76]. A complex composed of a second peptide and AgNPs was then captured by the peptides in the SAM by means of host–guest interactions. In the absence of MMP-2, AgNPs produce a drastic output in square wave voltammetry (SWV). On the other hand, when MMP-2 is present, the first peptide in the SAM is cleaved, releasing the second peptide functionalized with AgNPs from the electrode surface, resulting in a weaker current response (Figure 2a). A LOD of 0.12 pg/mL has been estimated. 

Aside from methods that use nanomaterials, various forms of amplified biodetection signals are obtainable. Radical polymerizations reactions, such as reversible addition-fragmentation chain-transfer (RAFT) polymerization and atom transfer radical polymerization (ATRP), are interesting implementations in biosensing platforms. Kim and Sikes recently wrote an exhaustive review that presents the principles of these reactions, comparing them in terms of performance and ease of application [77]. 

Hu and coworkers [65] managed to immobilise a recognition peptide via a Au–S bond on Au electrodes (Figure 2b). This peptide acted as proteolytic substrate to assess the MMP-2′s activity in a signal-on strategy. The grafting of ferrocenyl (Fc) polymers through electrochemically mediated RAFT (eRAFT) polymerization was responsible for the efficient accumulation of a vast quantity of Fc redox reporters on the electrode surface, lowering the LOD to 0.27 pg/mL under optimal conditions. Good performance was also demonstrated in complex serum samples and in the presence of interferents for the evaluation of selectivity. The authors also presented a similar method based on RAFT polymerization for the signal-amplified determination of thrombin activity, a serine protease routinely checked for in the diagnosis of coagulation disorders [78]. 

Working again with MMP-2 as analyte, Wang et al. instead used the electrochemically mediated ATRP (eATRP) reaction in a signal amplification strategy with ferrocenylmethyl methacrylate monomers to obtain a chain-growth polymer of Fc as an electroactive probe [79]. A specific peptide used as a recognition substrate is cleaved in the presence of MMP-2, hence lowering the peak current of Fc measured with SWV. A LOD of 0.53 fM was obtained under optimal conditions. A potential application in human serum was also verified by running experiments with spiked human serum, which were diluted 1000-fold, with recoveries ranging from 97% to 112%. 

Applying a very similar eATRP-based approach, Hu et al. targeted a different protease, namely the prostate-specific antigen (PSA), down to femtomolar concentrations [80]. As one of the most studied biomarkers for prostate carcinoma, PSA, a glycoprotein with endopeptidase activity, is always widely discussed in clinical research [81]. A quick view at the number of cases and victims of prostate cancer per year enlightens the importance of early diagnosis, given that the vast majority of patients rarely show symptoms before the latest stages of the disease [82]. In recent years, several peptide-based biosensors for the highly sensitive detection of PSA have been developed, harnessing different detection and amplification techniques, and exploiting the cleavage activity of the protease. As for MMPs, nanocomposites are widely used materials to enhance the sensitivity of the PSA detection mechanism. Meng et al., for instance, proposed the combination of graphene oxide and AgNPs on a Au electrode (Figure 2c) [83]. The use of DNAzymes (i.e., G-quadruplets/hemin, which possesses peroxidase-like activity) may also provide a dramatic boost to the sensor’s sensitivity. A few interesting applications of hemin/G-quadruplex DNAzymes coupled to peptides as recognition substrates for PSA detection have been reported in the recent literature [66,84].

Proteases represent markers for a variety of other applications. For instance, proteases produced from bacteria can be used to identify the presence of bacteria. Indeed, specific (synthetic) peptide sequences may find application as recognition receptors in the quantitative/qualitative detection and monitoring of different bacteria. Thus, peptide-based biosensors may constitute a cost-effective and rapid system for protection against pathogenic bacteria. With this in mind, Eissa and Zourob [85] worked on the multiplexed detection of *Listeria monocytogenes* (LOD of 9 CFU/mL) and *Staphylococcus aureus* (LOD of 3 CFU/mL) by exploiting the proteolytic activities of proteases produced from these two bacteria to hydrolyse a synthetic peptide sequence used as substrate. Magnetic nanoparticles were used to immobilise the peptide, harnessing the streptavidin–biotin interaction. An increased reduction in the peak current of [Fe(CN)_6_]^3-/4-^ was found in SWV when, due to the cleavage of the peptide sequence operated by the specific enzyme for each bacteria, the peptide/magnetic nanobeads were pulled away from the surface of AuNPs-modified screen-printed carbon electrodes (SPCEs). Selectivity experiments were also carried out.

Often, magnetic beads (MBs) offer a simple and practical solution for sample treatment and management. MBs are usually made of a thin polymer shell coupled to a dispersion of magnetic materials, presenting superparamagnetic properties. In the presence of a magnetic field, they exhibit magnetic properties; this effect ceases totally when the magnetic field is removed. These characteristics make MBs very useful for many different applications [86], including purification processes in bioanalytics; instead of centrifugation, filtration, or precipitation procedures, a simple magnet is sufficient. MB-assisted electrochemical biosensors pave the way for fast and handy protocols by decreasing electrode biofouling [87,88]. The immobilisation of peptide sequences on MBs merges the advantages of the abovementioned biosensors with peptide-based platforms. The attachment of a short peptide sequence labelled with biotin and fluorescein isothiocyanate (FITC) onto neutravidin-modified MBs recently allowed for the detection of an important protease in human cell lysates that finds clinical applications: trypsin, which is considered a biomarker for multiple forms of cancer when upregulated [89]. Together with other serine proteases, it activates other proteases (i.e., the abovementioned MMPs), thus inducing a proteolytic cascade, resulting in a decisive phase of tumour progression. Upon peptide digestion by trypsin, the modified MBs were enzymatically labelled with Fab fragments from anti-FITC antibodies conjugated with horseradish peroxidase (HRP). After being magnetically captured on the surface of unmodified SPCE, amperometric detection was conducted, which relies on the hydroquinone (HQ)/HRP/H_2_O_2_ system. Trypsin was successfully detected in cell lysates from pancreatic cancer, cervix carcinoma, and kidney cells, discriminating between pancreatic and nonpancreatic cancer cells.

An alternative to nanostructured macroscopic electrodes could be microelectrodes [90]. Pt-based microelectrodes of 25 µm diameter were recently applied by Mount’s group for the detection of trypsin in a peptide-based biosensor, demonstrating their applicability for the determination of clinically relevant concentrations of proteases [17].

As mentioned in the introduction of this paragraph, kinases are the second most important group of enzymes analysed with these catalytic peptide-based biosensors. In the case of kinase activity, detection is based on the determination of the phosphorylation process of the peptide used as substrate. In particular, kinases use adenosine 5′-triphosphate (ATP) as a phosphate group donor to catalyse the (reversible) phosphorylation of the hydroxyl groups of specific amino acid residues (i.e., serine, tyrosine, and threonine) in the peptide/protein sequence. This event is usually followed by conjugation with a signal reporter. Indeed, this kind of approach requires a more complex design compared with that of cleavage-based sensors for proteases. Likely due to this reason, and to the best of our knowledge, very few papers have been published in recent years on the detection of kinase activity. Cyclic adenosine monophosphate (cAMP)-dependent protein kinase and, more commonly, protein kinase A (PKA) indicate, when oversecreted, several pathological conditions including Alzheimer’s disease, cancer, diabetes, cardiovascular diseases, and others. A noteworthy example of its detection is the use of the aforementioned RAFT polymerization process to augment sensitivity, employing the peptide sequence LRRASLGGGGC as the recognition substrate [91,92].

Table 1 summarises various characteristics of the biosensors reported in the prior section, including remarks, performance, and whether or not an analysis on real samples was performed.

## 6. Peptides as Bioreceptors in Affinity Biosensors

**Figure 3:**
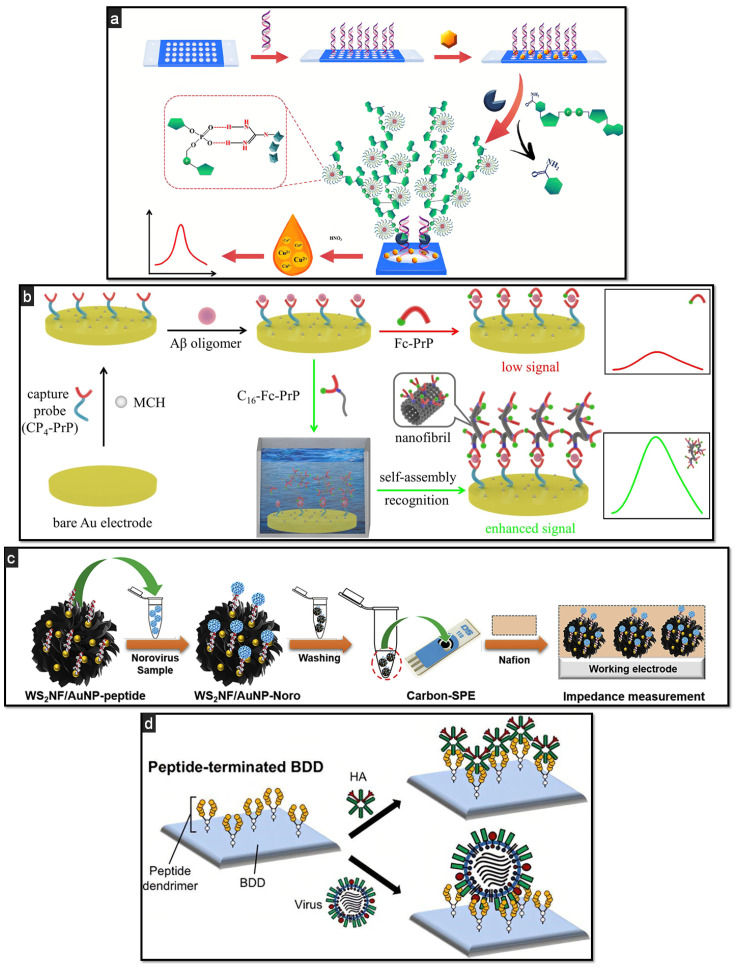
Schematic representation of some examples of peptide-based affinity platforms: (**a**) peptide-templated CuNPs as a probe for the detection of PARP-1, recognising and labelling PAR (PARP-1 catalysed form) by covalent-like interactions between guanidine groups and phosphate groups of PAR; quantitative determination via trace-level stripping voltammetry, reprinted with permission from [95]; (**b**) signal amplification strategy based on in situ peptide self-assembly for the detection, through SWV, of Aβ oligomer by designing a sandwich with a prion protein residue (PrP) that worked as a capture and Fc-conjugated signalling probe, reprinted with permission from [106]; (**c**) design of the label-free detection scheme based on peptide-functionalised WS_2_NF/AuNPs for norovirus through EIS analysis, reprinted with permission from [115] (**d**) HA and avian influenza virus immobilisation at BDD electrodes in a Fc-labelled approach, reprinted with permission from [116] Copyright (2020) American Chemical Society.

Cancer diagnosis is a vast clinical field, constantly enriched with novel biomarkers enabling discrimination between healthy and ill subjects, as well as between precancerous conditions and early stages of the disease. Many of these biomarkers are specific to certain types of tumours [93], while others may help to individuate the onset of different cancer-related processes and carcinogenesis. The anterior gradient proteins (AGR), for instance, are a family of proteins overexpressed in various human cancers. Ostatná et al. studied extensively the anterior gradient-2 (AGR2) oncoprotein with label-free current chronopotentiometry stripping (CPS) analysis, which allowed for the discrimination between specific and nonspecific interactions of AGR2 with a peptide aptamer [94]. Another promising tumour-related biomarker is poly(ADP-ribose) polymerase-1 (PARP-1). Wang et al. proposed an interesting method of labelling the peptide probe that was not based on unspecific electrostatic interactions [95]. In particular, they used peptide-templated copper nanoparticles (CuNPs) as a probe and harnessed specific covalent-like interactions between the guanidine groups of the probe and the acidic phosphate groups of PARP-1 (Figure 3a). This method allowed them to avoid unspecific interactions and achieve a low LOD of 0.004 U. Other interesting uses of metal nanoparticles such as AgNPs as labels can be found in the recent cancer-related literature [96].

Globally, colorectal cancer is the third most common type of cancer with an incidence of 10% of all cases [97]. Early diagnosis can truly constitute a life-saving step with this tumour. However, the gold standard technique for early detection, colonoscopy, is often despised by the vast majority of patients. That is why other less-invasive approaches are highly desirable and have already been utilized. Attractive analyses, for instance, are the determination of carcinoembryonic antigen (CEA) levels [98] and cytosensing of circulating tumour cells [99]. However, some of these methods often cannot discriminate between precancerous adenomas and carcinomas. An interesting exception was recently published concerning the development of an electrochemical affinity biosensor that uses synthetic peptides, designed using in silico modelling [100]. Such peptides were covalently immobilised on a Au electrode using benzoquinone through a surface chemistry approach. The detection of the leucine-rich α-2-glycoprotein-1 (LRG1) biomarker made possible the identification of the adenoma to carcinoma transition in 100-fold diluted human serum.

Interactions between proteins and metallic ions play a pivotal role and are very frequent at the biological and biochemical levels, defining structures and functions of proteins in the organism. Therefore, it is easy to imagine that peptides may share this kind of interaction with metallic ions, thus representing good recognition elements in peptide-based metallic ion sensing platforms [101]. With this in mind, Zhang and coworkers recently studied colorectal cancer-related oxidative stress under inflammatory conditions by investigating protein function and detecting proteins and their metal ion cofactors using peptide probes [102]. The cellular response to oxidative stress, indeed, is highly linked to the translocation of proteins and metal ions, as well as oxidative modifications of their functions and interactions.

Besides cancer, neurodegenerative disorders are among the most serious and debilitating diseases, responsible for heavy social and economic burdens. The two most prevalent are Alzheimer’s disease (AD) and Parkinson’s disease [103]. AD is strongly related to the aggregation of amyloid-β (Aβ) in the form of insoluble plaques in the brain, causing memory loss and dementia. The determination of Aβ in biological fluids such as blood, plasma, serum, and (above all) cerebrospinal fluid can give vital information for the (early) diagnosis of AD. For this reason, several peptide-based methods that aim at the detection of this biomarker have emerged over the years, for instance, by analysing Aβ using nanostructured platforms [104] or collecting platelets via peripheral blood sampling [105]. In a recent publication, this was accomplished by capturing the Aβ oligomer in a sandwich formed of two peptides, one as the capture probe, immobilised on a Au electrode, and the other, functionalized with Fc, as the label [106]. This latter peptide, after binding to the biomarker, was able to self-assemble on the surface of the electrode, generating an interesting form of signal amplification due to the large number of Fc moieties accumulated (Figure 3b).

Peptide-based biosensors have also been employed in the detection of biomarkers for other conditions and pathophysiological processes [107], including rheumatoid [108,109] and juvenile idiopathic [15] arthritis and acute kidney injury [110]. Furthermore, some peptide sequences for the affinity recognition of proteases have been selected. An efficient strategy to convert the binding event into a measurable electrochemical output is the conjugation of the peptide with a redox probe. For example, concerning the MMPs previously introduced, an uncommon approach was followed by Ma et al. for the detection of MMP-14: comparing a label-based method to a label-free one in an affinity biosensor instead of employing the previously mentioned catalytic approach [111]. In particular, a specific peptide that recognises the hemopexin domain PEX-14 of the protease, namely peptide ISC, was used either unlabelled or tagged with a Fc redox reporter. Both versions were immobilised on Au electrodes. However, the label-free architecture exhibited a signal-on trend, in which the EIS semicircle radius increased together with the concentration of MMP-14, whereas the Fc-labelled architecture showed a signal-off behaviour in DPV. The latter architecture performed slightly better, achieving a LOD of 0.3 pg/mL, compared with the LOD of 0.03 ng/mL obtained by the former one. Subsequently, a similar example was published by the same group using different peptides [112].

Apart from direct self-assembly, peptides demonstrate great compatibility with alkanethiol SAM, allowing for the design of peptide-based platforms on gold by covalent immobilisation of the recognition layer. This is the case for the sensing platform reported by Kim et al. [113] for the determination of nonstructural 1 protein, a biomarker of dengue virus. This simple architecture allows for the detection of the analyte via SWV and ESI in the presence of a redox probe with a LOD of 1.49 μg/mL. A similar sensing platform was designed for monitoring human norovirus in various food samples [114]. Here, eight different peptides were screened and compared after immobilisation at portable gold screen-printed electrodes via thiol SAM. The resulting portable impedimetric sensor allowed for discrimination among the sequences and selection of the one with better performance in terms of LOD. This was further applied as a bioreceptor in another electrochemical sensor based on tungsten disulfide nanoflowers decorated with gold nanoparticles [115]. The nanohybrid material functionalized with the peptide was incubated with oyster samples spiked with the analyte (Figure 3c). After sizing the norovirus, the biohybrid composite was separated from the sample solution, rinsed, and immobilised at carbon screen-printed electrodes with the help of Nafion ionomer. This strategy prevents the adsorption of interfering agents and is suggested when working with complex matrices, such as oyster samples.

Matsubara et al. [116] developed a tunable peptide-based biosensing platform for the determination of the antiviral nucleoprotein antibodies of Avian influenza, anti-hemagglutinin (HA), and several virus variants (from H1N1 and H3N2 to H5N3, H7N1, and H9N2), as illustrated in Figure 3d. The success of this double-analyte sensing relies on its biorecognition layer made of dendrimers bearing pentapeptides sequences able to recognize the receptor-binding site of all HA variants. In this example, the low steric hindrance of the peptide branches is instrumental to the recognition of both analytes, while the possibility to directly immobilise the dendrimers at boron-doped diamond (BDD) electrode surfaces assures high control and reproducibility of the modification itself. After characterisation with voltammetric techniques, the analytical protocol was defined using EIS as the detection technique. Apart from viruses, peptide-based sensors were also designed to monitor bacteria and toxins such as *Escherichia coli* (*E. coli*), *Staphylococcus aureus* (*S. aureus*), and *Salmonella typhimurium* (*S. typhi*) [117] or endotoxin [118].

## 7. Conclusions and Outlook

The synthetic nature, stability, and high yield of production of peptides are all very interesting properties for their use in different fields of science, especially in clinical diagnostics. An additional advantage is that different detection schemes are possible by using peptides; they can be used as substrates of proteases and kinases, which are enzymes considered to be important biomarkers of many different diseases. Furthermore, on the basis of their secondary structures, peptides can be used as affinity molecules for the specific binding of different analytes. Due to their physicochemical properties, peptides have also been proposed as antifouling molecules. Many examples of all-in-one multifunctional molecules with antifouling and biorecognition properties on the same peptide have also been proposed and reviewed in this manuscript. Despite these interesting properties, several issues remain—mainly, the total number of peptide sequences enabling recognition discovered until now is still significantly lower than that of antibodies.

In addition, peptides have been proposed as structural materials [2] and many examples are reported in the literature. Due to their tunable physicochemical properties, peptides are able to fold in compact structural motifs, shaping nanosized architectures in fibres, micelles, tubes, monolayers, bilayers, and strips that can have many different applications, including in vivo diagnostics.

The advantages of peptides versus antibodies in biosensing are evident in terms of their cost efficiency, high yield, and easier chemical synthesis. By contrast, it is hard to compare nucleic acids, including nucleic acid aptamers, and peptides. Peptides possess a variety of functional groups not present in nucleic acids (including nucleic acid aptamers) that can both enhance interactions, and thus affinity, with the target analyte and explain their functional and structural properties. Moreover, peptides have a different acid-base behaviour than nucleic acids. Thus, a comparison of these classes of interesting molecules in biosensing is not a trivial matter. Different considerations have to be kept in mind when making this comparison, such as selection or production procedures, which can lead to different evaluations. Therefore, it is not easy to predict which molecule is the best for a particular application. However, from the perspective of a researcher, it is important to have many different structural and functional molecules in order to better solve different bioanalytical issues and to increase the selectivity or multiplexing properties of the developed biosensors.

## Figures and Tables

**Table 1:** Catalytic peptide-based biosensors for the detection of various proteases and kinases. GCE = glassy carbon electrode; NHS = normal human serum; SA = sodium alginate; EIS = electrochemical impedance spectroscopy; PEI = polyethyleneimine; PtNTs = platinum nanotubes; CFU = colony-forming unit; HNE = human neutrophil elastase.

Target	Remarks	Target Peptide Sequence	Electrode	LOD	Real Samples	Ref.
MMP-2	Grafting of ferrocenyl polymers through eRAFT polymerization. Signal-on sensor. Fc as redox reporter.	PLGVR	Au electrode	0.27 pg/mL	/	[65]
MMP-2	Signal amplification via Au@Pt (nanorods) bimetallic nanozyme. H_2_O_2_-free peptide biosensor.	PLGVR	GCE	0.18 ng/mL	100-fold diluted human serum	[75]
MMP-2	Signal amplification via eATRP reaction and Fc polymers as electroactive probe. Measurements in SWV: Fc signal is decreased when the peptide used as recognition substrate is cleaved by MMP-2.	CGAPLGVRGA	Au electrode	0.53 fM	1000-fold diluted NHS	[79]
MMP-2	Signal amplification by anodic stripping of AgNPs. A first peptide is anchored onto the Au electrode and second-peptide-templated AgNPs are used to generate the signal. MMP-2 cleaves the first peptide, lowering the signal.	PLGVR	Au electrode	0.12 pg/mL	Human serum	[76]
MMP-7	JR2EC peptide as substrate. DPV analysis. Label-free approach: current increases linearly at higher concentrations of MMP-7 due to the cleavage of the peptide, which gives an electron transfer hindering effect (less surface area available).	/	CNTs/AuNPs/Au electrode	6 pg/mL	Spiked undiluted synthetic urine and 100-fold diluted human serum	[72]
MMP-7	Dual-reaction enhanced sensitivity. Amperometric detection. PdNPs catalytic probes combined with Au-rGO/methylene blue-SA nanocomposite.	KKKRPLALWRSCCC	GCE	3.1 fg/mL	Spiked healthy human serum	[69]
MMP-14	MMP-14-mediated cleavage of a Fc-carrying peptide placed on a Au electrode.	CPLPLRSWGLK	Au electrode	0.1 ng/L	Breast cancer cell lines type MCF-7	[70]
PSA	Signal amplification via eATRP reaction and Fc polymers as electroactive probe.	CSGGSSHSSKLQKK	Au electrode	3.2 fM	NHS	[80]
PSA	Signal-on biosensor based on peptide-conjugated hemin/G-quadruplex DNAzyme and rosebud-like MoSe_2_@rGO nanocomposite.	HSSKLQ	GCE	0.3 fg/mL	Clinical serum samples	[66]
PSA	Aggregation of silver ions and formation of AgNPs on a GO-modified Au electrode. If PSA is cleaved, the immobilisation of graphene oxide and the formation of AgNPs will not occur, hence leading to a subsequently decreased electrochemical response.	CGGHSSKLQFWYFWY	Au electrode	0.33 pg/mL	Spiked healthy human serum	[83]
PSA	Peptide-hemin/G-quadruplex conjugate. GCE modified with PEI-rGO@PtNTs nanocomposites. In the presence of PSA, the peptide-DNAzyme conjugate is cleaved, reducing the electrochemical signal.	CAAAHHHHHHHSSKLQ	GCE	2 fg/mL	Spiked 100-fold diluted human serum	[84]
Proteases from *L. monocytogenes* and *S. aureus*	Magnetic beads/peptide immobilised on an array of AuNPs-modified SPCE. Increased SWV reduction peak of ferro/ferricyanide when the cleaved magnetic beads/peptide were pulled away from the electrode surface.	*S. aureus*: ETKVEENEAIQK; *L. monocytogenes*: NMLSEVERE	AuNPs-modified SPCE	3 and 9 CFU/mL for *S. aureus* and *L. monocytogenes*, respectively	/	[85]
Trypsin	Amperometric detection of trypsin activity using the HQ/HRP/H_2_O_2_ system, and a peptide-sequence immobilised onto neutravidin-modified magnetic beads, dually labelled with biotin and fluorescein isothiocyanate.	FRR	SPCE	7 nM	HEK293T, HeLa, BxPC3 and PANC-1 cell lysates	[89]
Trypsin	Evaluation of Pt-based microelectrodes in a peptide-based biosensor for the detection of trypsin, envisaging a possible implantable application.	FRR	Pt microelectrode	2.9 nM	/	[17]
Trypsin	NiCo_2_O_4_ nanosheets and g-C_3_N_4_ nanocomposite for signal amplification.	CAGRAAADAD	GCE	10^−10^ mg/mL	10-fold diluted healthy human serum	[71]
Thrombin	RAFT polymerization as signal amplification. Recruitment of a large quantity of Fc tags on the electrode surface.	CGLVPRGS	Au electrode	2.7 µU/mL	Spiked NHS	[78]
HNE	HNE-mediated peptide cleavage leads to the release of a redox-labelled probe fragment, resulting in a measurable decrease of the electrochemical output via SWV. Immobilisation of a methylene blue-labelled peptide sequence.	APEEIMRRQ	Polycrystalline Au electrode	4 nM	Human blood	[64]
PKA	RAFT polymerization as signal amplification. Recruitment of a large quantity of Fc electroactive probes to each phosphorylated site.	LRRASLGGGGC	Au electrode	1.05 mU/mL	HepG2 cell lysates	[91]
PKA	eRAFT polymerization as signal amplification. Recruitment of a large quantity of Fc electroactive probes to each phosphorylated site.	LRRASLGGGGC	Au electrode	1.02 mU/mL	/	[92]
